# Ophthalmology

**Published:** 1899-03

**Authors:** Alvin A. Hubbell

**Affiliations:** Buffalo, N. Y., Clinical professor of ophthalmology and otology at the University of Buffalo


### OPHTHALMOLOGY.

CONDUCTED BY ALVIN A. HUBBELL, M. D., Buffalo, N. Y.,
Clinical professor of ophthalmology and otology at the University of Buffalo.

### ACCOMMODATION IN THE LOWER ANIMALS.

THEODORE BEER (*British Medical Journal*, November, 1898.) has made an interesting contribution to the somewhat neglected department of comparative pathology, in a paper on the act of accommodation of the eyes, which he has studied in a vast number of the lower animals. It occurs only in those which have eyes constructed on the principle of the camera obscura; in facetted eyes, on the other hand, thickness of retina in proportion to their total size is so great as to render accommodation unnecessary. Dr. Beer's methods were mainly three: ophthalmoscopic examination, measurement with the ophthalmometer, and electric stimulation of freshly excised eyes placed in normal saline solution at the body temperature. He finds that aquatic animals, with highly developed eyes, such as cephalopods and bony fishes, have eyes which are normally adapted to near vision and which undergo active accommodation for distance, the round lens being brought nearer the retina without changing its shape. The mechanism by which this is effected is different in the two cases. In the former there is a circular muscle attached to the front of the bulb and to the equator of the lens, and an elastic portion of the eye wall, which yields to the increased intraocular pressure when the muscle contracts; in the fishes there is a small muscle behind the lens (retractor lentis) which actually draws it toward the retina. The eyes of the terrestrial vertebrates are normally adapted for distant vision, and an active process of accommodation is required in looking at near objects. This act of accommodation is effected in one of two ways: in amphibia and snakes the unaltered lens is carried away from the retina; in the rest there is a change in its curvature. In every class of animals, with the exception of cattle, fishes and birds, there are certain species whose eyes do not possess the power of accommodation. Some of these are nocturnal in their habits; when their eyes are exposed to light the pupils are contracted to a linear form, and a kind of accommodation is thus effected. To others, such as the rabbit, observation of movement is more important than that of form, and hence the rabbit's power of altering its lens curvature is on a par with a man's ability to move his ears. Moreover, cave-dwelling and subterranean animals have but limited visual power, and do not need the faculty of accommodation. No animal can see equally well in air and water without an act of accommodation; aquatic animals are extremely short-sighted when on land, terrestrial very far-sighted in the water. A very few amphibiotic animals, which hunt both in land and water, have such an extraordinary range of accommodation that they can see near objects clearly under water. Finally, Dr. Beer has shown that animals which accommodate by alterations in the distance of the lens from the retina have an advantage over those which accommodate by changing the shape of the lens, in that age does not render them presbyopic.

### OPACITY OF THE CORNEA.

BERRY, G. A., Edinburgh, (*Edinburgh Med. Jour.*, April, 1898; *Amer. Jour. of the Med. Sciences*, December, 1898,) recommends for the treatment of opacities of the cornea, due to recent exudation, massage of the cornea. This is best done through the lid. The lid is rubbed quickly and with gradually increasing force over the cornea for half a minute or more at a time, once or twice daily. To do this effectually the rubbing must give rise to a moderate degree of surrounding hyperemia. The massage may be combined with the use of iodine ointment, or a good, and not too strong, stimulating effect may be secured by the daily use of the following prescription :

℞ Olei terebinth . . . . . . . . . . . . . . . . . . . . . . ʒj.

Olei amygd . . . . . . . . . . . . . . . . . . . . . . . . ʒij.

---

### THE USE OF ICHTHYOL IN DISEASES OF THE EYE.

EBERSON, M., (*Klinisch-therapeutische Wochenschrift*, 1898, No. 18, S. 669 ; *Amer. Jour. of the Med. Sciences*, December, 1898,) makes use of a 30 to 50 per cent. aqueous solution to which a small percentage of glycerin is added, and of a 5 per cent. ointment. Fifteen instances of its use are reported. The conclusions are (1) That it is a sure remedy for the cure of trachoma in that the course of the disease is shortened and the surfaces become smooth; (2) That this method is especially to be recommended for children; (3) That it quickly cures catarrhal conjunctivitis with or without corneal complications; (4) That it is a powerful remedy for clearing up corneal scars.

